# Supplementary material for: Comprehensive Annotation and Functional Exploration of MicroRNAs in Lettuce
Source: Front Plant Sci. 2021 Dec 24;12:781836. doi: 10.3389/fpls.2021.781836 (PMC8739914; doi:10.3389/fpls.2021.781836)
Supplement: Supplementary file 2 [file Data_Sheet_8.ZIP › GSM2230750.plot/Lsa-miR159a_Lsat_1_v5_gn_4_12381.1_621_TPlot.pdf]

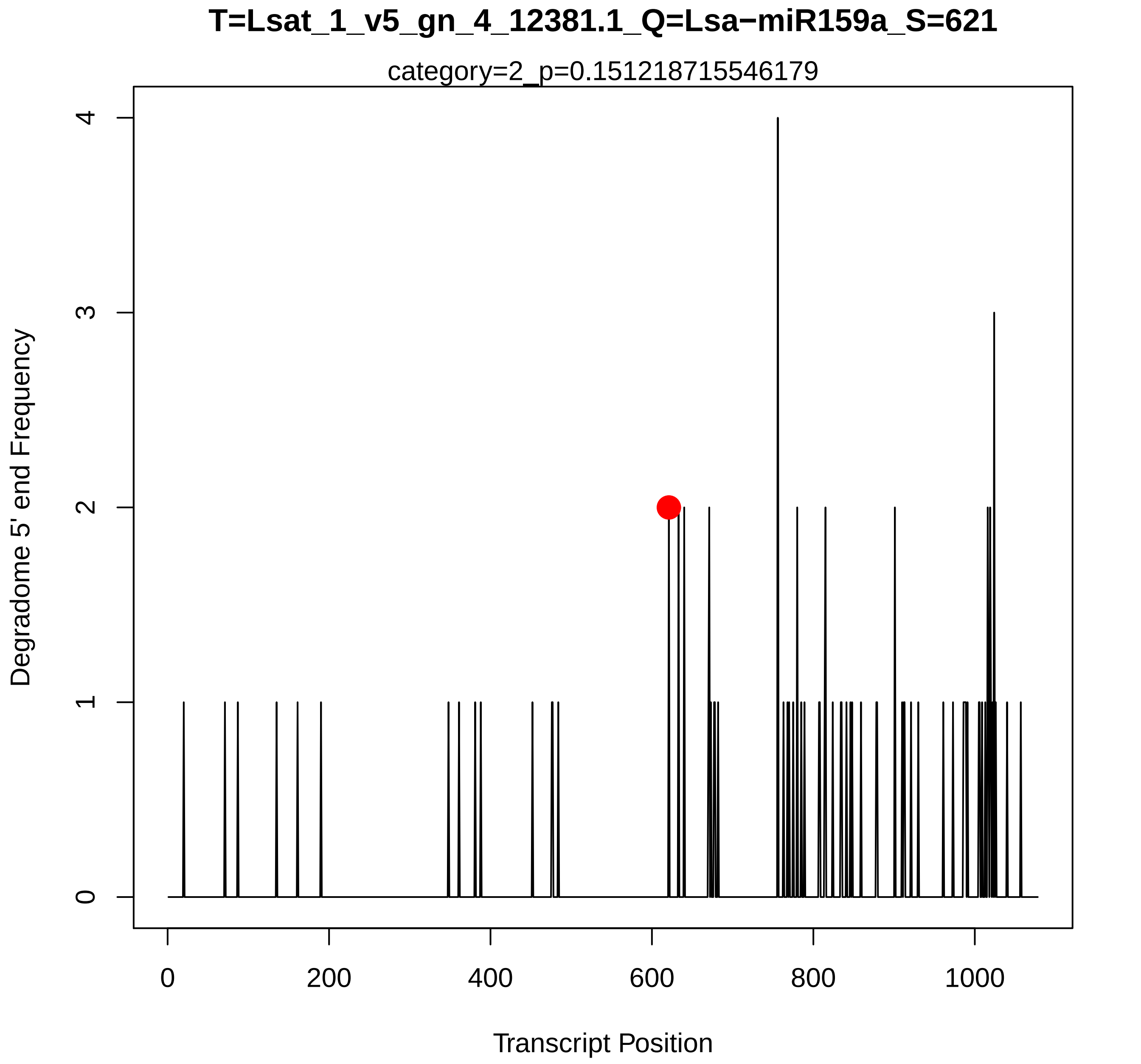

T=Lsat_1_v5_gn_4_12381.1_Q=Lsa−miR159a_S=621
category=2_p=0.151218715546179
Degradome 5' end Frequency
0
1
2
3
4
0
200
400
600
800
1000
Transcript Position
